# A Case of Lenvatinib-Induced Focal Segmental Glomerulosclerosis (FSGS) in Metastatic Medullary Thyroid Cancer

**DOI:** 10.1155/2018/6927639

**Published:** 2018-10-03

**Authors:** Kathryn Fleming, James McGuinness, David Kipgen, Hilary Glen, Pavlina Spiliopoulou

**Affiliations:** ^1^Beatson West of Scotland Cancer Centre (BWoSCC), Glasgow, UK; ^2^Queen Elizabeth University Hospital, Glasgow, UK; ^3^Imperial College London, Hammersmith Hospital, London, UK

## Abstract

We describe a case of lenvatinib (E7080) associated focal segmental glomerulosclerosis (FSGS) encountered during the treatment of metastatic medullary thyroid cancer. Proteinuria is a relatively common side effect of VEGF-targeted treatments and can occasionally result in treatment discontinuation. Here, we describe a patient who developed secondary FSGS necessitating discontinuation of treatment at first but who was subsequently rechallenged with anti-VEGF-targeted treatment without recurrence of proteinuria. Lenvatinib was a novel experimental agent at the time the treatment took place; however, its recent licensing for the treatment of thyroid malignancies in the UK makes reporting of these adverse effects all the more important now.

## 1. Introduction

Angiogenesis is an imperative process for the survival and growth of cancer cells with the most important mediator of angiogenesis being vascular-endothelial growth factor (VEGF) and its associated downstream effectors—VEGF receptors (VEGFRs). These can normally be found on the surface of endothelial cells and endothelial cell precursors, fibroblasts, and macrophages as well as renal epithelial cells such as podocytes [1–3].

VEGF is produced in large amounts by the glomerular podocyte and guides endothelial cells in the developing kidney. Moreover, in adult life, VEGF maintains integrity of the glomerular filtration barrier [4–7]. VEGF/VEGFR axis can be therapeutically targeted either with antibodies aimed at the soluble extracellular ligands or with small molecule inhibitors (tyrosine kinase inhibitors, TKIs) which block the intracellular domains of VEGFRs. Bevacizumab, a humanized monoclonal antibody against VEGF-A is approved for use in combination with chemotherapy in patients with metastatic colorectal cancer and ovarian cancer [8, 9]. Aflibercept (a recombinant fusion protein that blocks the activity of VEGF-A, VEGF-B, and placental growth factor PlGF) has also been approved for use in patients with metastatic colorectal cancer [10]. Small molecule tyrosine kinase inhibitors, pazopanib and sunitinib, are both approved for use as first-line treatment in metastatic renal cell carcinoma and inhibit, amongst others, VEGFR1-3, PDGFR*α*/*β*, and the receptor c-KIT [11, 12]. Sorafenib is currently used in advanced hepatocellular carcinoma [13] whereas regorafenib in colorectal and gastrointestinal stromal tumours [14, 15].

## 2. Case Report

In June 2002, a 36-year-old woman presented to her primary health care doctor with a history of flushing, diarrhoea, night sweats, and a clinically detectable mass in her left medial supraclavicular fossa. Her past medical history consisted only of essential hypertension for which she did not require prescribed therapy. Her family history included a brother with a diagnosis of sarcoma and two other non-first-degree relatives with primary brain malignancies.

Fine needle aspiration confirmed the diagnosis of medullary thyroid cancer, and in July 2002, she underwent total thyroidectomy with left-sided modified radical neck dissection and central compartment clearance. At this point, concerns were raised regarding optimal cytoreduction as the appearances of the central compartment, level 4 and level 5 nodes, were that of extensive disease. In order to maximise local disease control, she received adjuvant radical radiotherapy delivering 60 Gy to the thyroid bed.

Two years after completion of treatment, in February 2004, follow-up repeat imaging reported a recurrent nodule at level 4 of her neck. Subsequent resection confirmed this to be recurrent medullary thyroid cancer with no evidence of distant spread at the time. She continued to be monitored at the oncology clinic and remained disease-free until four years later, in May 2008, when computer tomography (CT) imaging revealed new pulmonary parenchymal metastases. These were closely monitored for the next 2 years with repeat imaging and measurement of calcitonin levels. In May 2010, it was decided that the patient should embark on systemic anticancer treatment.

She was offered participation in a phase 2 clinical trial with the agent lenvatinib (E7080) and she commenced treatment with 24 mg once daily in May 2010. One week into therapy, it was noted that she was marginally hypertensive with a blood pressure of 140/100 mmHg. No proteinuria was identified at this point, but she was commenced on 5 mg of amlodipine to manage hypertension. Monitoring of blood pressure and urinalysis continued as per study protocol.

After two completed cycles of lenvatinib, CT imaging reported a reduction in size of all lesions. Further tumour assessment after 4 months confirmed a partial response to treatment with a 50% reduction of the sum of the long diameters of target lesions. She was experiencing various grade 1 toxicities throughout this time but was keen to maintain treatment given the good response. Due to the multiple low-grade toxicities, the dose of lenvatinib was initially reduced to 20 mg and thereafter to 14 mg.

In December 2011, 19 months after starting lenvatinib, she developed mild ankle oedema. Urinalysis carried out at the time identified proteinuria. A subsequent 24-hour urine collection identified 3.1 g/litre of proteinuria, equating to a urinary protein creatinine ratio (UPCR) of 625. The patient had not started any other medications and the incidence of proteinuria was felt to be lenvatinib related. Treatment with lenvatinib was ceased; however, due to concerns regarding possible intrinsic renal disease, she underwent screening for glomerulonephritis which was negative.

**Figure 1:**
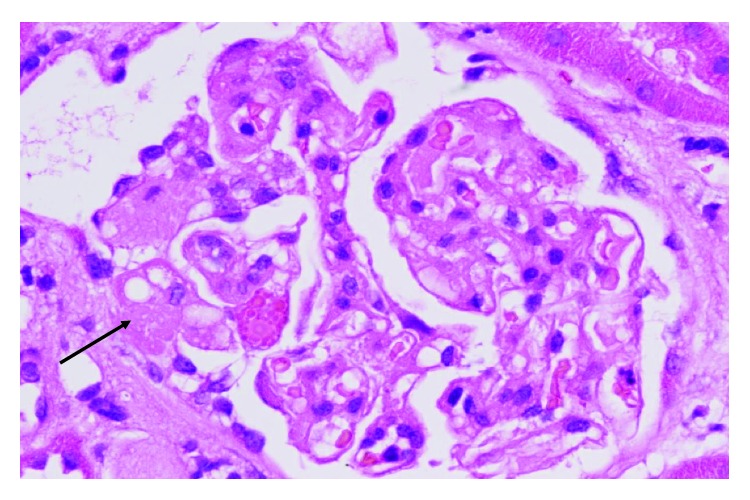
Glomerulus showing segmental sclerosis (arrow). H + E, ×40.

**Figure 2:**
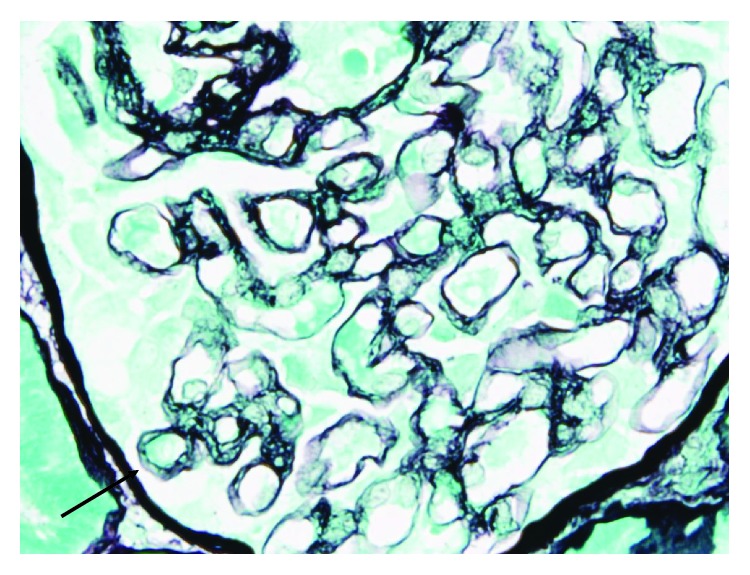
Glomerulus showing segmental splitting of capillary walls (arrow). PA Meth Ag, ×60.

**Figure 3:**
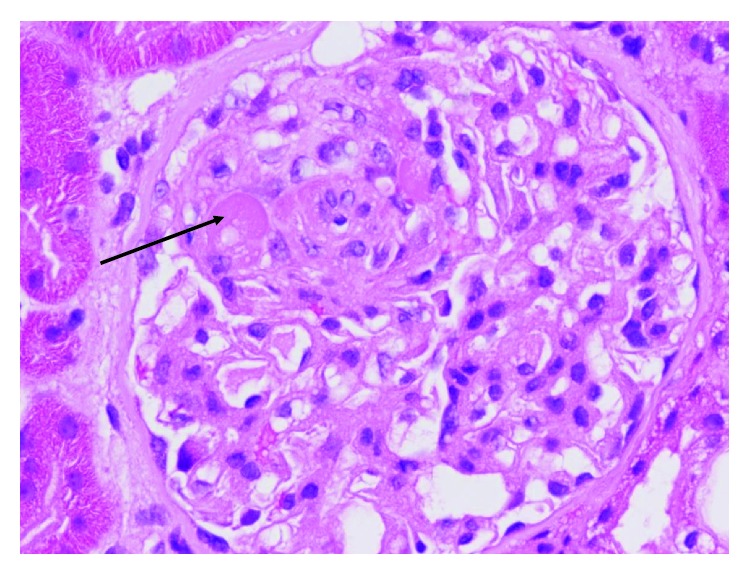
Glomerulus showing segmental hyalinosis (arrow). H + E, ×40.

A subsequent renal biopsy showed focal segmental glomerulosclerosis (FSGS) in two of twelve viable glomeruli, with tuft-capsule adhesion, hyalinosis, segmental intracapillary hypercellularity, and segmental splitting of capillary walls, predominantly in regions of segmental sclerosis. There was mild tubular atrophy, interstitial fibrosis, mild/moderate arterial intimal fibroelastic thickening, and mild arteriolosclerosis. Immunofluorescence showed no staining in glomeruli. Electron microscopy showed mild patchy reduplication of the basement membrane and effacement of only 20% of podocyte foot processes. There were no widespread electron dense deposits and no endothelial cell tubule-reticular inclusions. The endothelial cells showed no evidence of activation or damage. The appearances were consistent with a diagnosis of focal segmental glomerulosclerosis (FSGS). The lack of widespread podocyte foot process effacement suggests a secondary form of FSGS, which in the context of anti-VEGF treatment, could be mediated by microangiopathy. Whilst there was no histological evidence of acute thrombotic microangiopathy, it is possible that some of the pathological changes seen (splitting of glomerular capillary walls and mild arteriosclerosis) could be related to chronic low-grade endothelial cell damage. Based on histology, it is not possible to be certain whether the FSGS was caused by direct podocyte injury or whether it was related to endothelial cell injury. Histological slides are illustrated below in Figures 1, 2, and 3.

Throughout this time, excretory function remained stable. Treatment with an ACE-inhibitor (ACEi) was introduced but due to poor tolerance and the quick improvement of the proteinuria after cessation of lenvatinib, the ACEi was stopped and patient's blood pressure was monitored closely.

**Figure 4:**
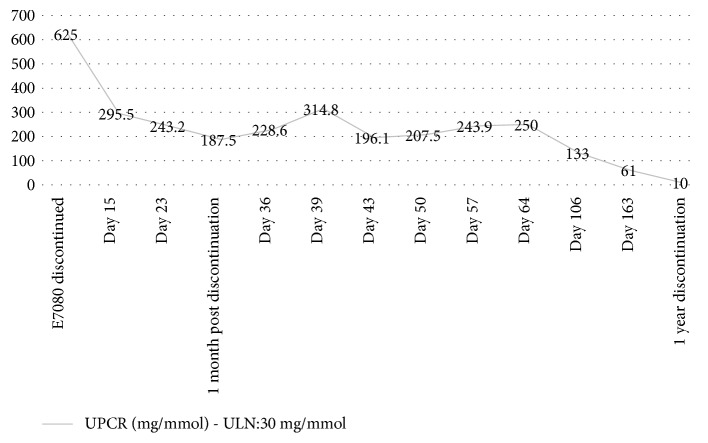
Withdrawal of lenvatinib and subsequent improvement of UPCR.

Withdrawal of lenvatinib had a marked effect on the levels of proteinuria, as illustrated in Figure 4.

The patient continued follow-up at the renal clinic on a regular basis until July 2013, when she was discharged with no evidence of proteinuria, normotensive and with normal excretory renal function.

After her discharge from the renal clinic and between 2013 and 2017, the patient was treated with vandetanib, nintedanib, and cabozantinib with no evidence of recurrent renal disease.

### 2.1. Lenvatinib (E7080)

E7080, also known as lenvatinib, is a potent inhibitor of the receptor protein kinases VEGFR-2 and VEGFR-3 but also displays inhibitory binding properties against VGFR-1, FGFR-1, and PDGFR*α*/*β*, albeit at significantly higher IC_50_ (half maximal inhibitory concentration, IC_50_). Its ability to restrain angiogenesis was shown on human umbilical vein endothelial cells (HUVEC) where E7080 inhibited VEGFR-2 phosphorylation and thereby capillary tube formation [16]. Apart from angiogenesis, E7080 decreased lymphangiogenesis in both the primary tumour of human breast adenocarcinoma cells in xenografts as well as in metastatic nodules in the lymph nodes of nude mice bearing these tumours [17]. Glen et al. showed in preclinical experiments that abrogation of FGFR and PDGFR signalling by E7080 inhibited invasion and migration of human melanoma cells lines (DX3) and human osteosarcoma epithelial cells (U2OS) [18]. Its potency against FGFR-1 differentiates E7080 from other currently approved tyrosine kinase inhibitors with antiangiogenesis properties [16, 19].

The preclinical data above were confirmed in several early phase human trials with E7080 in 2011 and 2012 in US, Europe, and Japan. Whilst establishing pharmacokinetic and pharmacodynamic properties of the drug, safety and preliminary efficacy was also well described. Lenvatinib was well tolerated at doses from 10 mg BID to 25 mg OD [20–24] and was associated with a reduction in disease activity biomarkers [23], partial response, and stable disease according to response evaluation criteria in solid tumours [21]. These findings were further established in phase 2 trials and notably responses were demonstrated in thyroid cancer [25–27].

Approval in thyroid cancer was granted in light of significant improvement in progression-free survival (PFS) compared with placebo in patients with radioiodine-refractory differentiated thyroid cancer in a phase 3 study (SELECT study) [28]. Lenvatinib improved median PFS over placebo by almost 15 months (HR 0.21; *p* < 0.01) and induced an objective response rate of 64.8%. The median survival results were diluted due to crossover of the patients from the placebo arm to the treatment arm; nevertheless, a subgroup analysis on patients stratified by age showed that older patients (>71 years old) had a survival advantage when treated with lenvatinib compared to placebo (HR, 0.53; *p* = 0.02), and the younger subgroup achieved a PFS of 20.2 months versus 3.7 m (*p* < 0.001) [29].

### 2.2. VEGF-Mediated Renal Toxicity

Proteinuria and hypertension are the two most commonly reported side-effects of VEGF inhibitors and frequently the cause for therapy discontinuation. Proteinuria is used as a surrogate marker for glomerular damage and hypertension often accompanies and aggravates this.

The pathophysiology of proteinuria and glomerular damage in anti-VEGF therapy remains complex and far from well understood. Biopsy-proven cases of glomerular disease in anti-VEGF therapy are few; however, most have demonstrated changes in keeping with glomerular thrombotic microangiopathy (TMA) histology, with predominant endotheliosis and membranoproliferative changes [30, 31]. Other histological changes documented include cryoglobulinaemic glomerulonephritis, acute interstitial nephritis, collapsing and crescentic glomerulonephropathies, and FSGS plus TMA [32–35].

It has been theorized that hypertension is caused by decreased vascular production of nitrous oxide induced by inhibiting VEGF. This leads to renal haemodynamic compromise and subsequent proteinuria (much akin to exercise-related proteinuria) [30]. However, a mouse model study showed that glomerular injury preceded hypertension [36] and many cases document glomerular injury in the absence of hypertension [37], indicating that it cannot be the only trigger for proteinuria in anti-VEGF treated patients.

Inhibition of VEGF in podocytes (by injection of anti-VEGF antibodies or VEGF gene deletion) results in loss of endothelial fenestrations in glomerular capillaries, proliferation of glomerular endothelial cells, loss of podocytes, and proteinuria in mice [4, 5]. VEGF appears to be a crucial endothelial survival factor and its inhibition often manifests as TMA, a histology strikingly similar to that of severe preeclampsia—as placenta overproduces a soluble VEGF receptor (fms-like tyrosine kinase 1) that acts as a VEGF antagonist.

Izzedine et al.'s 8-year follow-up study results from 2014 shed great light in anti-VEGF-related renal injury. It showed that in 100 patients who developed renal disease whilst on anti-VEGF treatment, the main histology associated with TKIs was minimal change disease and/or collapsing-like focal segmental glomerulosclerosis (MCN/cFSGS), a FSGC variant which is considered a separate entity to FSGS. In the same analysis, TMA histology was most frequently associated with VEGF-ligand targeted therapy (such as bevacizumab and aflibercept) suggesting two, possibly distinct pathophysiologies [37, 38] between renal damage caused by targeting the VEGF ligand as opposed to targeting the VEGFR tyrosine kinase domain. This could potentially be explained by considering the associations and signal transduction pathways between podocytes, endothelial cells, and VEGF. Podocytes produce vascular endothelial growth factor (VEGF), whereas VEGF receptor tyrosine kinases (RTKs) are expressed by both podocytes and glomerular endothelial cells.

Our case demonstrates a secondary form of FSGS pathology which cannot confidently be attributed to TMA but could potentially represent the end result of chronic low-grade endothelial cell damage. The moderate histological findings were in keeping with a less-severe clinical course of the FSGS, with fast resolution of proteinuria and hypertension. More significantly, rechallenging the patient with additional three agents blocking the VEGF axis did not result in recurrence of the renal damage.

## 3. Conclusion

Fortunately, as in our case, cessation of anti-VEGF inhibitors in most cases significantly improves proteinuria and hypertension. In practice, in patients with dipstick detectable proteinuria and BP >130/80, ACE inhibitors are often utilised [30]. In most cases where ACE inhibition fails to control proteinuria, anti-VEGF therapy is ceased. It is interesting to note that a drug holiday followed by reinitiation of anti-VEGF therapy might not result in recurrence of proteinuria [39] which was the experience with our patient. At present, there is unlikely to be any other way of safely continuing anti-VEGF therapy where there is significant proteinuria with or without hypertension (such as immunosuppressive therapy as may be done for other causes of TMA and FSGS). Our understanding of the mechanisms of damage needs to be improved before we can safely continue anti-VEGF treatment in the presence of proteinuria without compromising the prognostic outcome of patients with renal syndromes secondary to anti-VEGF treatment [40, 41].
